# *METTL3* Regulated the Meat Quality of Rex Rabbits by Controlling *PCK2* Expression via a YTHDF2–N6-Methyladenosine Axis

**DOI:** 10.3390/foods11111549

**Published:** 2022-05-25

**Authors:** Gang Luo, Tongyan Zhu, Zhanjun Ren

**Affiliations:** College of Animal Science and Technology, Northwest A&F University, Xianyang 712100, China; luogang66@nwafu.edu.cn (G.L.); zhutongyan234@nwafu.edu.cn (T.Z.)

**Keywords:** Rex rabbits, m^6^A methylation, *PCK2* gene, meat quality, flavor and taste

## Abstract

N6-methyladenosine (m^6^A) is the most prevalent internal mRNA modification in eukaryotes. The M^6^A modification plays an important role in transcription and cell function. The mechanism by which m^6^A modification regulates meat quality remains elusive. In this study, gene knockout and overexpression were used to explore m^6^A-modified regulation of meat quality. The content of PCK2 in blood increased significantly with the increase of Rex rabbits’ age. *PCK2* expression levels in the longissimus lumborum and liver also increased significantly with the increase of Rex rabbits’ age. However, the expression level of *PCK2* showed no significant difference in adipose tissue. In cell experiments, we found that *METTL3* inhibited adipocyte differentiation by targeting the *PCK2* gene via the recognition function of *YTHDF2*. Finally, the results of correlation analysis showed that *PCK2* expression was positively correlated with intramuscular fat, whereas *PCK2* expression was negatively correlated with total water loss rate at three different stages. In addition, *PCK2* expression was also negatively correlated with reduced pH value at 75 and 165 days. Intramuscular fat content, pH and muscle water holding capacity are the main factors affecting the taste and flavor of muscle. Therefore, N6-methyladenosine regulated muscle quality by targeting the *PCK2* gene.

## 1. Introduction

Rabbit meat has been widely accepted as a healthy food worldwide due to its high nutritional value and easy digestibility, which make it especially suitable for children and elderly people. It contains not only a high content of protein, essential amino acids, mineral and trace element components such as selenium and cobalt but also a low fat and cholesterol level [1]. However, the low intramuscular fat content reduced rabbit meat consumption and hindered the development of the rabbit industry. Appropriately increased IMF (intramuscular fat) content can improve the meat quality, including color, tenderness, flavor and juiciness [2,3,4,5]. In addition, greater intramuscular fat can lead to an increased postmortem pH [6]. Besides, studies on pork showed that the higher the pH value, the smaller the cooking loss [7]; the pH value has a significant effect on the tenderness, color, water retention and moisture distribution of muscle fibers [8]. Thus, the formation of fat has a great impact on the taste, flavor and juiciness of the muscle. Obesity or adipogenesis is characterized by the process of cell proliferation and differentiation at the cellular level. Therefore, since the manipulation of adipocyte differentiation could be a promising strategy for the flavor improvement of rabbit meat, improving our understanding of the molecular mechanism of adipogenesis is of major significance.

N6-methyladenosine (m^6^A) occurs in the sixth nitrogen atom of the RNA adenylate and is the most abundant internal modification in eukaryotic mRNAs [9]. M^6^A is installed by a multicomponent methyltransferase complex consisting of methyltransferase-like 3 (METTL3), *METTL14* and Wilms tumor 1–associated protein (WTAP) and erased by m^6^A demethylase fat mass and obesity-associated protein (FTO) [10]. *YTHDF2* act as the m^6^A “readers” that can promote the translation of mRNAs and mediate m^6^A-dependent RNA degradation [11]. In addition, m^6^A modification also plays a key role in biological processes such as cellular differentiation, lipid accumulation and energy metabolism [12,13,14]. However, the detailed regulation mechanism of m^6^A modification is unknown.

Most of what is known about the regulation and function of phosphoenolpyruvate carboxykinase (PEPCK-C) comes from studies in rats and mice, where its exclusive function is gluconeogenesis [15]. *PCK2* encodes phosphoenolpyruvate carboxykinase 2 and participates in the metabolic pathway of gluconeogenesis [16,17]. Overexpression of *PCK2* increased gluconeogenesis and free fatty acid (FFA) reesterification [16]. The results showed that the expression of *PCK2* was significantly correlated with the content of polyunsaturated fatty acids (PUFAs), which affected the oxidative sensitivity of meat [18]. *PCK2* has a direct repetitive sequence with the AGGTCA motif and can bind to peroxisome-proliferator-activated receptor gamma (PPARγ) [19]. Peroxisome-proliferator-activated receptor-γ (PPARγ) and members of the CCAAT/enhancer-binding family of proteins (C/EBPα) play a significant role in the regulation of adipogenesis [20,21]. These results suggest that *PCK2* may regulate fat deposition. In addition, the expression of *PCK2* was positively correlated with the content of IMF [22], which suggested that *PCK2* may participate in the regulation of IMF. However, the regulation of *PCK2* in adipogenesis is poorly understood.

In the present study, the content of PCK2 in the blood and the expression levels of the *PCK2* gene in the longissimus lumborum and liver tissues increased significantly with increasing age in Rex rabbits. However, the expression level of the *PCK2* gene remained almost unchanged at different stages. Subsequently, we found that the loss of *METTL3* promoted expression of the *PCK2* gene via the recognition function of *YTHDF2*. In addition, *PCK2* can promote adipocyte differentiation. Finally, the results of correlation analysis showed that the correlation coefficients between *PCK2* expression in the longissimus lumborum and intramuscular fat content in 35-day-old, 75-day-old and 165-day-old rabbits were 0.94 (*p* < 0.01), 0.85 (*p* < 0.05) and 0.82 (*p* < 0.05), respectively. The expression level of the *PCK2* gene was negatively correlated with the total water loss rate, and the correlation coefficient were 0.87 (*p* < 0.05), 0.79 (*p* < 0.05) and 0.74 (*p* < 0.05) in 35-day-old, 75-day-old and 165-day-old rabbits, respectively. Reduced pH value was negatively correlated with *PCK2* expression only in 75-day-old and 165-day-old rabbits, and the correlation coefficient were 0.85 (*p* < 0.05) and 0.88 (*p* < 0.05), respectively. Intramuscular fat content, pH and muscle water holding capacity are the main factors affecting the taste and flavor of muscle [23]. Therefore, N6-methyladenosine affected the flavor of muscle by targeting the *PCK2* gene. This study provides a scientific, molecular theoretical basis for cultivating rabbits with good meat quality.

## 2. Material and Methods

### 2.1. Animals and Tissue Collection

Three 0-day-old, six 35-day-old, six 75-day-old and six 165-day-old female Rex rabbits were used in this study, which were raised under standard conditions at the Northwest A&F University farm (Yangling, Shanxi, China). All the experimental procedures in this study were approved by the guidelines of Animal Experiment Committee Northwest A&F University, China. Three replicates were selected for all cell experiments, and six replicates were selected for all tissue or blood experiments in this study.

### 2.2. Sample Collection

Before slaughtering, 5 mL of heart blood was collected from all rabbits except 0-day-old rabbits and placed at room temperature for 2 h. Serum was isolated and stored at −80 °C. After slaughtering, the required amount of the left perirenal fat, the longissimus lumborum and the liver were quickly separated and immediately cooled in liquid nitrogen. All samples were transferred to a −80 °C refrigerator after half an hour. Meat samples required for meat quality determination were separated as required and determined at an appropriate time.

### 2.3. Cell Culture and Transfection

The perirenal fat of 0-day-old rabbits was isolated immediately after slaughter and placed in PBS containing 4% double antibody. Subsequently, the fat was digested with 0.1% collagenase type I (GIBCO, Carlsbad, CA, USA) in a water bath at 37 °C, and the growth medium (DM/F12, 10% fetal bovine serum, 2% penicillin-streptomycin) was used to culture preadipocytes. The cells were cultured in the incubator (5% CO_2_, 37 °C) using culture bottles after filtration with 70 and 40 nm cell sieves. Cell transfection was achieved by using Lipofectamine 2000 (Invitrogen, Carlsbad, CA, USA) for siRNA following the manufacturer’s protocols when cells grow to 80–90% in the culture bottle or cell dish. Then, an adipogenic cocktail (0.5 mM 3-isobutyl-1- methylxanthine, 10% fetal bovine serum, 1 µM dexamethasone and 1.7 µM insulin) was added into the growth medium to induce differentiation. After 3 days, the culture medium was changed to maintain differentiation. After another 3 days, the growth medium (DM/F12, 10% fetal bovine serum, 2% penicillin-streptomycin and 1.7 µM insulin) was changed to maintain differentiation.

### 2.4. RT-qPCR

Total RNA was extracted using TRIzol reagent (Invitrogen, CA, USA) and reverse transcribed into cDNA by the Prime Script RT Reagent Kit with genomic DNA Eraser (Takara Bio, Saint-Germain-en Laye, France). The QRT-PCR experiments were carried out with 2.5 ng/µL cDNA template, SYBR Premix Ex Taq II and Rox Plus and 10 pmol/µL forward/reverse primer on a Quant Studio^TM^ (Applied Biosystems by Thermo Fisher Scientific, Waltham, MA, USA). β-Actin was used as an internal control. All the primers used for qPCR are listed in Table 1.

### 2.5. Measurement of PCK2 in Blood

Concentrations of PCK2 were determined according to the manufacturer’s instructions using PCK2 ELISA kits (Ruixinbio, Quanzhou, China). The actual sensitivities of PCK2 were typically <0.1 ng/mL. This PCK2 ELISA kits only recognizes PCK2 and is not disturbed by other similar substances.

### 2.6. Gene-Specific m^6^A qPCR

Total RNA was extracted using TRIzol regent (Invitrogen, CA, USA). Then, the Magna MeRIP m^6^A Kit (Millipore) was used to examine m^6^A modifications on individual genes according to the manufacturer’s instructions. mRNA was purified by using mRNA miniprep kit (SIGMA, Cat. No. MRN10). Briefly, 0.5 μg of mRNA was removed and placed into a new microcentrifuge tube labeled “RNA input”. The input sample was then kept at −80 °C. The sample was later used for comparison in RT-PCR methods. Subsequently, RNase inhibitor and 5 × IP buffer were used to enrich the m^6^A modification in mRNA. Finally, PCR analysis was performed after elution with eluent. 

### 2.7. Western Blotting

Cells were harvested using RIPA lysis buffer (CWBIO, Jiangsu, China) containing protease inhibitor (CWBIO, Jiangsu, China) and incubated on ice for 30 min. Subsequently, WB was performed using the method used in the previous study [24]. The membranes were incubated with the following primary antibodies: PCK2 (Shenggong, Shanghai, China), METTL3 (Shenggong, Shanghai, China), FTO (Fitzgerald, Acton, USA) and β-actin (Absin, Shanghai, China). Finally, the images were obtained with a Bio-Rad GelDoc system equipped with a Universal Hood III (Bio-Rad, California, USA), and the integrated optical density (IOD) was calculated using Gel-Pro Analyzer 4.0.0.4. Actin was used as an internal control.

### 2.8. Measurement of Triglyceride Content and Oil Red-O Staining

The contents of triglyceride (TG) in adipocytes were measured using the TG Assay Kit (Applygen, Beijing, China) according to the manufacturer’s protocol. The method of oil red-O staining was the same as that used in previous articles [25]. Finally, oil red-O was eluted from the stained cells with isopropanol and quantified by measuring the optical density values at 510 nm wavelength.

### 2.9. Determination of Meat Quality Traits

Intramuscular fat was measured by Soxhlet extraction method [26]. Briefly, the meat sample was dehydrated to a constant amount in the oven and then crushed. Then, the sample weight, sample and paper package weight and the total weight after extraction were recorded as M1, M2 and M3, respectively. The formula for intramuscular fat content (w) is as follows: w = (M2 − M3)/M1 × 100%. The longissimus lumborum was used to measure the pH values at 45 min and 24 h after rabbits were sacrificed by an insert electrode pH-star according to Blasco’s method [23]. A meat moisture meter was used to determine the total water content according to the instructions. For the determination methods of the cooked meat rate and the drip loss, refer to the previous study [27].

### 2.10. Statistical Analysis

All data are presented as the mean ± standard deviation (SD). Differences in the mean values between 2 groups were tested for significance with a Student’s *t*-test, and between 3 groups, the significance was determined with one-way ANOVA using GraphPad Prism7 (GraphPad Software, La Jolla, CA, USA). *p* < 0.05 and *p* < 0.01 were deemed to be significant and highly significant, respectively. Correlation analyses were performed using SAS9.2 (Statistical Analysis System; SAS Institute Inc., Raleigh, NC, USA), the method used was Pearson’s correlation coefficient. Two-tail Student’s *t*-test was used to analyze the significance of the different levels. *p*-values of less than 0.05 and 0.01 were considered to be significantly and extremely significantly correlated.

## 3. Results

### 3.1. Analysis of the Content of PCK2 in Blood, the Expression of the PCK2 Gene and m^6^A Modification of the PCK2 Gene in Different Tissues of Rex Rabbits

**Figure 1:**
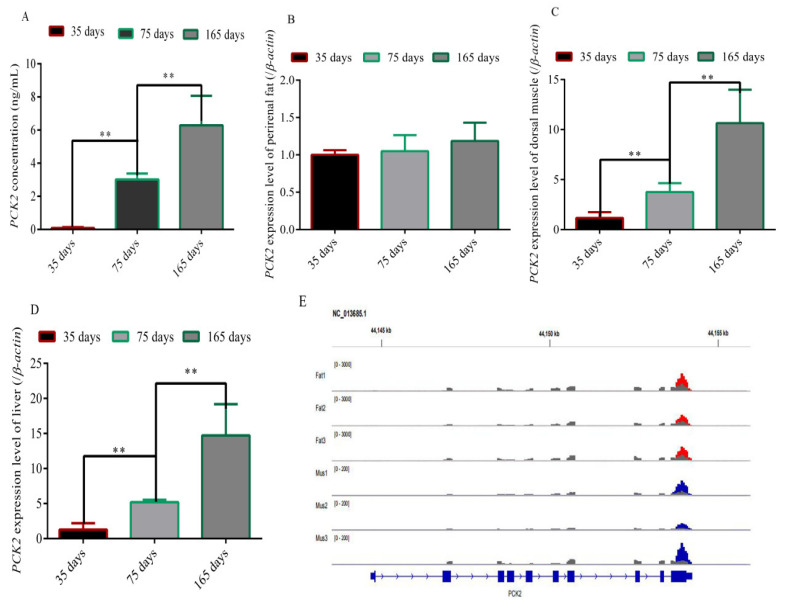
**The content of PCK2 in blood and the expression and methylation of the *PCK2* gene in various tissues**. (**A**) Content of PCK2 in Rex rabbit blood; (**B**) expression levels of *PCK2* in perirenal fat; (**C**) expression levels of *PCK2* in dorsal muscle; (**D**) expression levels of *PCK2* in the liver; (**E**) examples of dynamic methylation with m^6^A peaks in the *PCK2* gene. (“**”, *p* ≤ 0.01).

First, by measuring the content of *PCK2* in the blood of Rex rabbits at different stages, it was found that the PCK2 content increased with age (Figure 1A). To investigate whether the expression level of the *PCK2* gene is related to fat deposition, the expression levels of *PCK2* were detected in the perirenal fat, the longissimus lumborum and the liver of 35-day-old, 75-day-old and 165-day-old Rex rabbits. The results showed that the expression level of *PCK2* was not significantly different in the perirenal fat of different stages (Figure 1B), while the expression levels of *PCK2* in the longissimus lumborum and liver of old Rex rabbits were significantly higher than that of young Rex rabbits (*p* < 0.01) (Figure 1C,D). Finally, we detected the methylation of the *PCK2* gene in the longissimus lumborum and perirenal fat of newborn rabbits and found that the modification of the *PCK2* gene was significantly different between the longissimus lumborum and the perirenal fat (Figure 1E). Together, these results showed that the expression of the *PCK2* gene may be modified by m^6^A and related to fat deposition.

### 3.2. Knockout of YTHDF2 Gene Partially Restored the Effect of METTL3 Knockout on Adipocyte Differentiation

**Figure 2:**
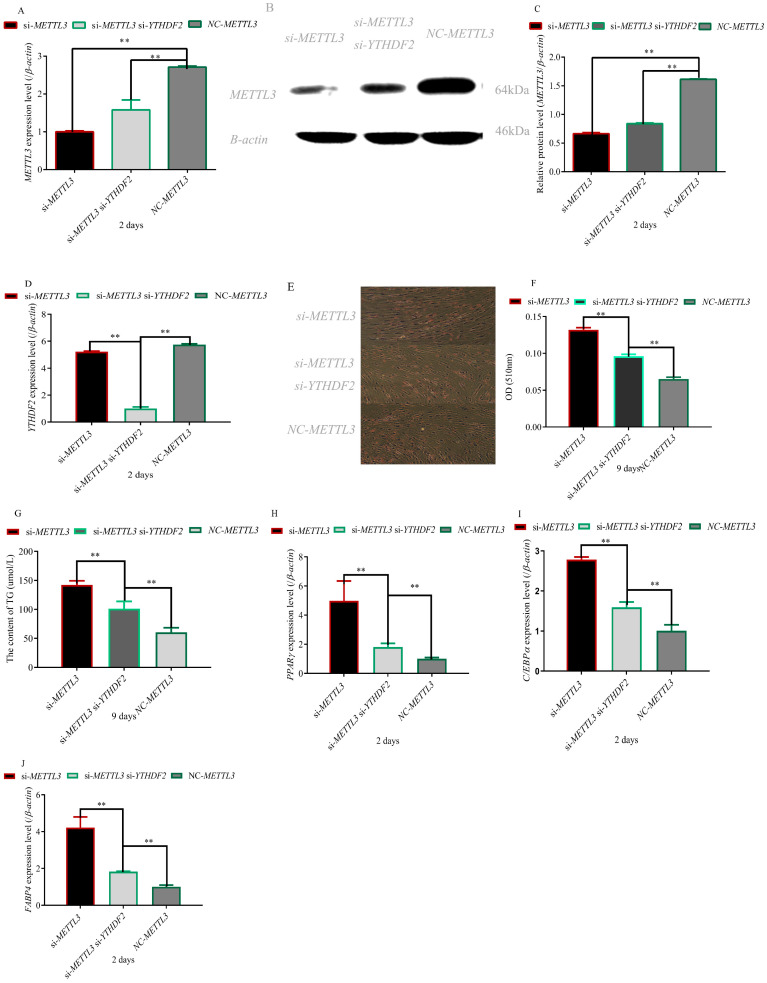
***METTL3*-regulated preadipocyte differentiation in an m^6^A–YTHDF2-dependent manner**. (**A**–**C**) *METTL3* expression level during preadipocyte differentiation after transfecting with *si-METTL3*, *si-YTHDF2* and NC; (**D**) *YTHDF2* expression level during preadipocyte differentiation after transfecting with *si-METTL3*, *si-YTHDF2* and NC; (**E**,**F**) results of oil red-O staining on day 9 after transfecting with *si-METTL3*, *si-YTHDF2* and NC; (**G**) triglyceride content on day 9 after transfecting with *si-METTL3*, *si-YTHDF2* and NC; (**H**–**J**) expression level of marker genes *PPARγ,* *CEBPα* and *FABP4* during preadipocyte differentiation after transfecting with *si-METTL3*, *si-YTHDF2* and NC (“**”, *p* ≤ 0.01).

To investigate the regulation of *YTHDF2* and *METTL3* on adipocyte differentiation, we simultaneously interfered with *YTHDF2* and *METTL3* in preadipocytes. The results indicated that *METTL3* and *YTHDF2* were successfully knocked out in the experimental group (Figure 2A–D). Knockout of the *METTL3* gene promoted the differentiation of preadipocytes. However, *YTHDF2* knockout inhibited the promoting effect of *METTL3* knockout on differentiation of preadipocytes. Downexpression of *YTHDF2* in *METTL3* knockdown preadipocytes significantly reduced the lipid droplets (*p* < 0.01) (Figure 2E,F), accompanied by significantly decreased TG content (*p* < 0.01) (Figure 2G). Similarly, knockout of *YTHDF2* partly decreased the expression of adipogenic marker *PPARγ*, *C/EBPa* and *FABP4* genes in *METTL3* knockdown preadipocytes (*p* < 0.01) (Figure 2H–J). Consistently, *YTHDF2* partly influenced the regulation of *METTL3* on the differentiation of preadipocytes.

### 3.3. Effect of METTL3 and YTHDF2 on the Expression Level of the PCK2 Gene

**Figure 3:**
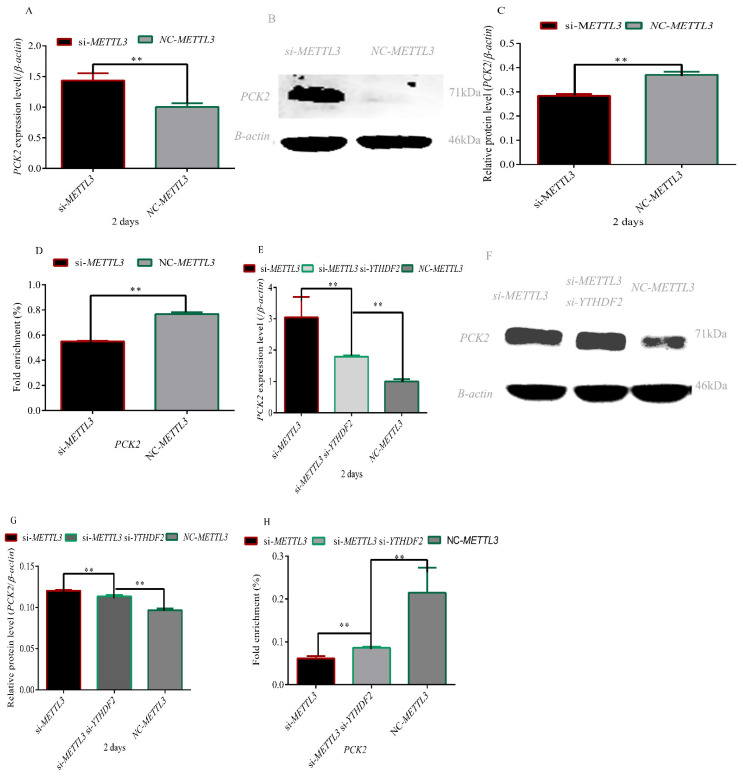
***METTL3* regulated the expression level of the *PCK2* gene in an m^6^A–YTHDF2-dependent manner.** (**A**–**C**) *PCK2* expression level during preadipocyte differentiation after transfecting with *si-METTL3* and NC; (**D**) fold enrichment of the *PCK2* gene after transfecting with *si-METTL3* and NC; (**E**–**G**) *PCK2* expression level during preadipocyte differentiation after transfecting with *si-METTL3*, si-*YTHDF2* and NC; (**H**) fold enrichment of the *PCK2* gene after transfecting with *si-METTL3*, si-*YTHDF2* and NC; (“**”, *p* ≤ 0.01).

To determine whether *METTL3* regulates the *PCK2* gene m^6^A–YTHDF2 dependently, we first detected the expression of *PCK2* in *METTL3* knockdown adipocytes and found that the expression levels of *PCK2* were significantly higher than those of the NC group (*p* < 0.01) (Figure 3A–C). However, the fold enrichment of *PCK2* methylation modification decreased significantly in the METTL3-depleted adipocytes (*p* < 0.01) (Figure 3D). Next, we assessed the role of *YTHDF2* by knocking down *METTL3* and *YTHDF2*. Knockdown of *YTHDF2* significantly decreased the mRNA and protein levels of the *PCK2* gene in *METTL3* knockdown cells (*p* < 0.01) (Figure 3E–G). In addition, knockdown of *YTHDF2* partly recovered the fold enrichment of *PCK2* methylation modification (Figure 3H). These results demonstrated that *METTL3* m^6^A–YTHDF2 dependently regulates the *PCK2* gene.

### 3.4. Effect of PCK2 on the Differentiation of Preadipocytes

**Figure 4:**
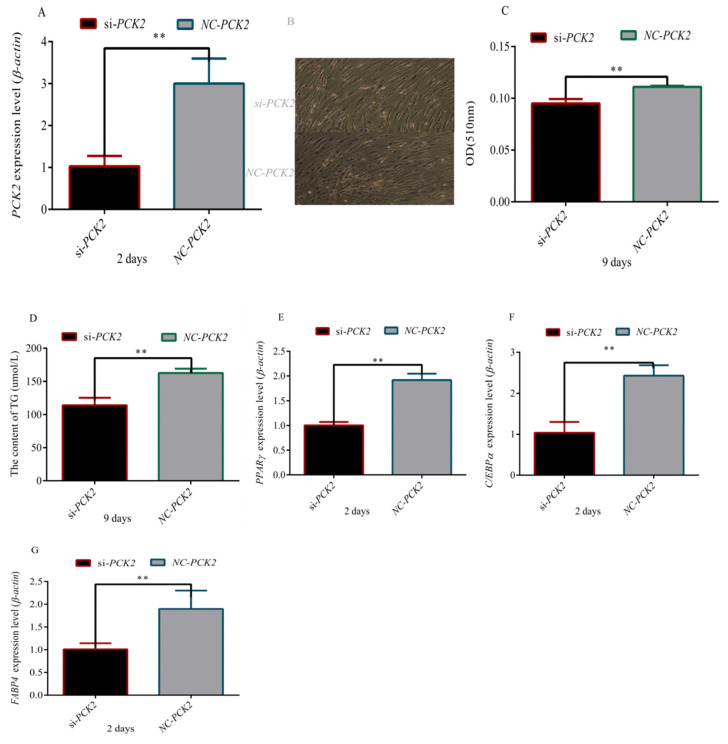
**Inhibition of *PCK2* inhibited rabbit preadipocyte differentiation**. (**A**) *PCK2* expression level during preadipocyte differentiation after transfecting with *si-PCK2* and NC; (**B**,**C**) results of oil red-O staining on day 9 after transfecting with *si-PCK2* and NC; (**D**) triglyceride content on day 9 after transfecting with *si-PCK2* and NC; (**E**–**G**) expression level of marker genes *PPARγ,* *CEBPα* and *FABP4* during preadipocyte differentiation after transfecting with *si-PCK2* and NC (“**”, *p* ≤ 0.01).

To understand the mechanism by which *PCK2* regulates the differentiation of preadipocytes, we first interfered with the *PCK2* gene in preadipocytes (Figure 4A–C). In *PCK2*-knockdown adipocytes, we found that the number and volume of lipid droplets was significantly reduced using oil red-O staining and determination of TG content (Figure 4D–F). At the same time, the expression of adipogenic marker *PPARγ*, *C/EBPa* and *FABP4* genes was lower in *PCK2* knockdown preadipocytes than those in the NC group (*p* < 0.01) (Figure 4G–I). Overall, *PCK2* played an important role in regulating the differentiation of preadipocytes.

### 3.5. Correlation Analysis between Expression Level of the PCK2 Gene in the Longissimus Lumborum and Meat Quality Traits

The correlation analysis is plotted in Table 2. The correlation coefficients between intramuscular fat content and *PCK2* expression in 35-day-old, 75-day-old and 165-day-old rabbits were 0.94 (*p* < 0.01), 0.85 (*p* < 0.05) and 0.82 (*p* < 0.05), respectively. Reduced pH value was negatively correlated with *PCK2* expression only in 75-day-old and 165-day-old rabbits, and the correlation coefficients were 0.85 (*p* < 0.05) and 0.88 (*p* < 0.05), respectively. The total water loss rate was negatively correlated with the expression level of the *PCK2* gene, and the correlation coefficients were 0.87 (*p* < 0.05), 0.79 (*p* < 0.05) and 0.74 (*p* < 0.05) in 35-day-old, 75-day-old and 165-day-old rabbits, respectively.

## 4. Discussion

In this study, we found that the level of PCK2 in blood also increased with age (Figure 1A). Previous studies showed that there was a positive correlation between fat deposition and age in growing rabbits [27]. It indicated that *PCK2* may regulate fat deposition. To investigate whether *PCK2* is associated with fat deposition, we detected the expression levels of *PCK2* in the perirenal fat, longissimus lumborum and liver of Rex rabbits and found that the expression level of *PCK2* increased with age in the longissimus lumborum and liver tissues (Figure 1C,D). However, there was no significant difference in the expression level of *PCK2* in the perirenal fat at different stages (Figure 1B). The liver is the main site of fat synthesis [28]. Due to the need of adaptability, many animals deposit a large amount of fat in the liver; the liver returns to its original state after the stored fat is consumed by energy supply, and the whole change process is reversible [29,30]. In addition, the study showed that the volume of adipocytes gradually increased and the rate of fat deposition increased rapidly in lambs entering the fattening stage [30]. These results further indicated that *PCK2* played an important role in fat deposition. At the same time, we found that the *PCK2* gene was modified by m^6^A in the longissimus lumborum and perirenal adipose tissues of Rex rabbits.

The profiles and function of METTL3-mediated mRNA m^6^A are different depending on the tissue and the developmental stage [31,32,33]. Indeed, it has also been reported that the expression of many genes is regulated by either *METTL3* or *FTO* alone [34]. A previous study also suggested the functional importance of the heterodimer formation of *METTL3* and *METTL14* in adipocyte differentiation [35].

In this study, we found that interference with METTL3 can promote the differentiation of adipocytes (Figure 2) and the expression level of the PCK2 gene (Figure 3A–C). *METTL3* inhibits hepatic insulin sensitivity and promotes fatty acid metabolism [36]. A loss of the initial insulin suppression of glucose output in vitro and an associated increased activity of hepatic *PCK2* has been reported [37]. It was recently demonstrated that hepatic *PCK2* mRNA expression increased following the induction of chronic hypoglycemia in fetal sheep and that this was associated with a decrease in circulating insulin and an increase in plasma cortisol [38]. *PPARγ* agonists may sensitize the response of peripheral tissues to insulin by inhibiting the expression of *11β-HSD1* in adipocytes [39]. *11β-HSD1*-knockout mice lost the ability to convert 11-dehydrocortisone into cortisol and inhibited the activity of *PEPCK* [40]. Cortisol has been shown to play a key role in the upregulation of hepatic *PCK2* activity and expression in the late-gestation fetus [41,42], so the increase in hepatic *HSDL1* mRNA expression and intrahepatic cortisol production may stimulate increased *PCK2* mRNA expression. RNA N6-methyladenosine methyltransferase *METTL3* facilitates colorectal cancer by activating the m^6^A–GLUT1–mTORC1 axis [43]. There is a positive correlation between the expression of *GLUT-1* and *PCK2* in laryngeal squamous cell carcinoma [44]. These results indicated *METTL3* can regulate the expression of the *PCK2* gene. However, the specific regulatory mechanism is still unclear. In this study, we found that mRNA and protein levels of the *PCK2* gene increased significantly, whereas m^6^A methylation of the *PCK2* gene decreased significantly after transfection with si-METTL3. Based on the above results, we inferred that *METTL3* regulated the expression level of *PCK2* by m^6^A methylation.

*METTL3*-mediated mRNA m^6^A modification can be recognized by the individual m^6^A reader proteins, which play key roles in controlling gene expression. Early studies showed that YTHDF2-binding increases mRNA decay [45]. In addition, other roles for these reader proteins are rapidly emerging in the regulation of specific mRNAs in different types of cells during different stimuli [46,47,48]. Moreover, the study revealed a novel mechanism wherein downregulated spinal cord *METTL3* in coordination with *YTHDF2* contributes to the modulation of inflammatory pain by stabilizing the upregulation of *TET1* in spinal neurons [49]. *METTL3* epigenetically repressed *YPEL5* in an m^6^A–YTHDF2-dependent manner by targeting the m^6^A site in the coding sequence region of the *YPEL5* transcript [50]. In this study, we found that *YTHDF2* silencing could partially reverse the phenotype induced by *METTL3* knockdown, including differentiation of adipocytes and methylation level of the *PCK2* gene (Figure 2, Figure 3). These results suggested *METTL3* regulates adipocyte differentiation by targeting the *PCK2* gene in an m^6^A–YTHDF2-dependent manner.

In order to explore the mechanism by which *PCK2* regulates adipogenesis, we knocked out the *PCK2* gene in adipocytes and found that the differentiation of adipocytes was significantly inhibited (Figure 4). It has been found that *PCK1* and *PCK2* can be used as candidate genes of obesity, which can participate in the synthesis of glycerophosphate and promote the accumulation of fat in human body [51]. In addition, the loss of *PCK2* in islets can damage insulin secretion, which suggested that the activity of *PCK2* is related to insulin secretion [52]. In the majority of these studies [53,54,55], the degree of obesity and not the direct measurement of insulin resistance was used to identify individuals as insulin resistant vs. insulin sensitive. *PCK2* was upregulated by effectors of this pathway by recruiting *ATF4* to a consensus *AARE* site located at the *PCK2* proximal promoter [56]. A clue to the mechanism for the integrative role of *PCK2* in cancer metabolism arises from its transcription regulation by *ATF4* under nutrient stress [57,58]. *ATF4* regulates age-related and diet-induced obesity as well as glucose homeostasis in mammals and has conserved metabolic functions in flies [59]. In addition, the expression level of *PCK2* was higher in Rex rabbits with morefat deposition. These results indicated that *PCK2* can promoted adipocyte differentiation.

Adipocyte differentiation is a necessary process of fat deposition. In order to explore the relationship between the expression level of *PCK2* and intramuscular fat, we analyzed the correlation between the expression level of *PCK2* and intramuscular fat and found that they are positively correlated (Table 2). Previous study has shown that *PCK2* can promote adipogenesis. In summary, *PCK2* can regulate the production of intramuscular fat. Studies have shown that intramuscular fat is positively correlated with the pH of muscle [60]. The faster the pH value drops, the faster the meat spoils. Therefore, a reduced pH value is an important indicator of good meat quality. In this study, we found that the expression level of *PCK2* was negatively correlated with reduced pH value in rabbits at 75 and 165 days of age. At the same time, there was a strong correlation between intramuscular fat and muscle system hydraulics [61]. Water in muscle also affects meat production. In this study, we found the expression level of *PCK2* was negatively correlated with the total water loss rate. Meat with low drip loss is more easily accepted by consumers [62]. A study reported that consumers are attracted to rabbit meat according to its healthiness, sensory properties and hedonistic quality (variability on visual appeal) [63].

Intramuscular fat, pH and muscle water holding capacity are important indicators affecting muscle flavor. At the same time, intramuscular fat is positively correlated with sensory tenderness, juiciness and flavor [64]. Therefore, it can be concluded that *PCK2* plays an important role in muscle flavor.

## 5. Conclusions

**Figure 5:**
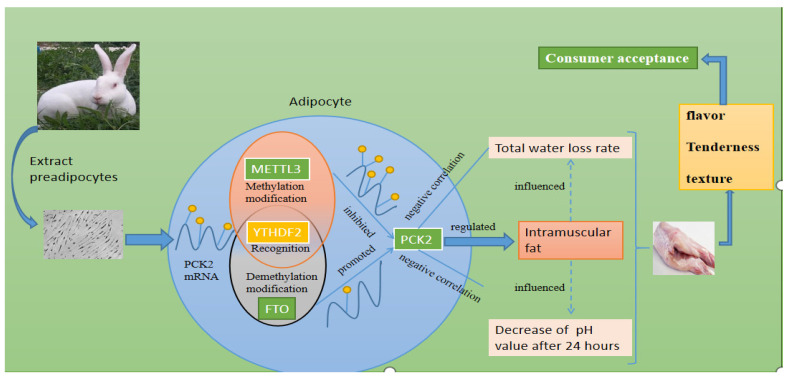
N6-methyladenosine affected the flavor and taste of muscle by targeting the PCK2 gene.

*METTL3* decreased the expression level of the *PCK2* gene in an m^6^A–YTHDF2-dependent manner. The expression level of the *PCK2* gene was negatively correlated with reduced pH value and total water loss rate. However, intramuscular fat was positively correlated with the expression of the *PCK2* gene. As shown in Figure 5, *METTL3* affects meat quality by regulating the expression of *PCK2.* The study provides a scientific, molecular theoretical basis for cultivating rabbits with good meat quality.

## Figures and Tables

**Table 1:** Primers used in this study.

Gene Name	Primer Sequence (5′-3′)	Tm (°C)	Product Size (bp)
*YTHDF2*	CAGACACAGCCATTGCCTCCAC	60	122
	CCGTTATGACCGAACCCACTGC		
*METTL3*	CCCACCTCAGTGGATCTGTT	60	189
	ACCCAGAGGAAGAGAAAGCC		
*PCK2*	AACAGGAGGTGCGTGACATT	60	250
	GGGACAGGGAGTGTGAGAAG		
*PPARγ*	GAGGACATCCAGGACAACC	61	168
	GTCCGTCTCCGTCTTCTTT		
*β-actin*	GGAGATCGTGCGGGACAT	61.4	318
	GTTGAAGGTGGTCTCGTGGAT		
*C/EBPα*	GCGGGAACGAACAACAT	64	172
	GGCGGTCATTGTCACTGGTC		
*FABP4*	GGCCAGGAATTTGATGAAGTC	61.4	140
	AGTTTATCGCCCTCCCGTT		
*si-YTHDF2*	CAUGAAUACUAUAGACCAATT		
	UUGGUCUAUAGUAUUCAUGTT		
*si-METTL3*	UCAAGGAACAACAGAGCAATT		
	UUGCUCUGUUGUUCCUUAGTT		
*si-PCK2*	GGGAACAGGAGGUGCGUGATT		
	UCACGCACCUCCUGUUCCCTT		
Negative Control	UUCUCCGAACGUGUCACGUTT		
	ACGUGACACGUUCGGAGAATT		

**Table 2:** Correlation analysis of expression level of the *PCK2* gene with meat quality traits in Rex rabbits.

Expression Level/Meat Quality Traits		IntramuscuLAR Fat Content	Cooked Meat Rate	Drip Loss	Reduced pH Value	Total Water Loss Rate
Expression level of *PCK2*	Age of 35 days	0.94 **	0.45	0.58	0.39	−0.87 *
	Age of 75 days	0.85 *	−0.46	0.21	−0.85 *	−0.79 *
	Age of 165 days	0.82 *	−0.04	−0.28	−0.88 *	−0.74 *

Note: Single asterisk (*) and double asterisks (**) indicate significant and highly significant correlations at the 0.05 and 0.01 probability levels, respectively.

## Data Availability

The datasets used and/or analyzed during the current study are available from the corresponding authors upon reasonable request.

## Abbreviations

m^6^A	N6-methyladenosine
NCD	non-communicable disease
PCK2	phosphoenolpyruvate carboxykinase 2
YTHDF2	YTH domain family 2
METTL3	methyltransferase-like 3
METTL14	methyltransferase-like 14
WTAP	Wilms tumor 1–associated protein
PPARγ	peroxisome proliferator-activated receptor-γ
C/EBPα	CCAAT/enhancer-binding family of proteins
FABP4	fatty-acid-binding protein 4
TG	triglyceride content
